# Effect of Deposition Temperature on Zn Interstitials and Oxygen Vacancies in RF-Sputtered ZnO Thin Films and Thin Film-Transistors

**DOI:** 10.3390/ma17215153

**Published:** 2024-10-23

**Authors:** Sasikala Muthusamy, Sudhakar Bharatan, Sinthamani Sivaprakasam, Ranjithkumar Mohanam

**Affiliations:** Department of Electrical and Electronics Engineering, Sri Venkateswara College of Engineering, Sriperumbudur 602117, India; msasikala@svce.ac.in (S.M.); sinthamani@svce.ac.in (S.S.); mranjith@svce.ac.in (R.M.)

**Keywords:** RF sputtering, ZnO, X-Ray diffraction, X-Ray photoelectron spectroscopy, thin-film transistor

## Abstract

ZnO thin films were deposited using RF sputtering by varying the argon:oxygen gas flow rates and substrate temperatures. Structural, optical and electrical characterization of ZnO thin films were systematically carried out using X-Ray diffraction (XRD), scanning electron microscopy (SEM), UV–visible spectroscopy, X-Ray photoelectron spectroscopy (XPS) and Hall measurements. Film deposited at room temperature and annealed at 300 °C exhibited low O_2_ incorporation with localized defects and a high percentage of Zn interstitials. A large crystalline size and fewer grain boundaries resulted in a high Hall mobility of 46.09 cm^2^/V-s Deposition at higher substrate temperatures resulted in improvement in O_2_ incorporation through the annihilation of localized defects and decrease in oxygen vacancies and Zn interstitials. Urbach tails within the bandgap were identified using the absorption spectrum and compared with the % defects from XPS. Bottom-gate thin-film transistors were subsequently fabricated on a SiO_2_/p-Si substrate using the combination of RF sputtering, wet etching and photolithography. Variation in the substrate temperature showed performance enhancement in terms of the leakage current, threshold voltage, sub-threshold swing and I_ON_/I_OFF_ ratio. Thin-film transistor (TFT) devices deposited at 300 °C resulted in an O_2_-rich surface through chemisorption, which led to a reduction in the leakage current of up to 10^−12^ A and a 10-fold reduction in the sub-threshold swing (SS) from 30 V to 2.8 V. Further TFT optimization was carried out by reducing the ZnO thickness to 50 nm, which resulted in a field-effect mobility of 1.1 cm^2^/V-s and I_ON_/I_OFF_ ratio of 10^5^.

## 1. Introduction

Enormous research efforts on metal oxide semiconductors have been carried out in the recent past, due to which, they have become promising candidates in the field of solar cells [1,2,3,4], thin-film transistors (TFTs) [5,6,7,8], photodetectors [9,10,11,12], memory [13,14,15], gas sensors [16,17,18,19], bio sensors [20,21,22] and complementary metal oxide semiconductor (CMOS) circuits [23,24]. Ease of availability of metal oxide proves to be a key attribute in realizing terrestrial thin-film solar cells, detectors and TFTs. Even though the efficiencies of metal oxide (ZnO and TiO_2_) thin-film solar cells are lower compared to perovskite solar cells (26.7% efficiency) [25], metal oxides play a vital role in improving the efficiency and chemical stability of solar cells. Electrical and optical properties such as a wide bandgap (3.37 eV direct bandgap in the case of ZnO) [26], high mobility, transparency, tunability and substrate compatibility make metal oxides a promising candidate in photodetector and gas sensing applications such as UV sensing, optoelectronics, imaging (medical imaging) and environmental monitoring [9,10,11,12].

In many of the traditional electronic applications, e.g., pixel and peripheral driver circuits, amorphous silicon (Si)-based TFTs are widely used due to their ease of integration with a complementary metal oxide semiconductor (CMOS). However, large transistor sizing limits the application of TFTs in integrated circuits [27]. Further, advancements in the fields of the Internet of Things (IOT), artificial intelligence (AI) and machine learning (ML) require large memory and high bandwidth, leading to the scaling down and 3D integration of devices. There have been reports on stacked In_2_O_3_ TFTs on a SiO_2_/Si substrate in a 3D integration form of devices [28]. Even though Si technology can easily be realized with advanced manufacturing processes, its performance suffers due to a high process temperature, poor mobility, bias stress, threshold voltage instability and reliability issues. Hence, metal oxide-based TFTs are the potential alternative to Si-based TFTs in the areas of flexible display, health care, environment and automotive applications. The process-level advantage of an oxide semiconductor is the ability to realize large-area device performance on any substrate at low process temperatures using a non-equilibrium deposition technique such as RF sputtering.

Among the various metal oxide semiconductor cations, Zn and Sn are considered to be non-toxic and abundantly available. ZnO exhibits a wide bandgap (3.37 eV) [26], low cost and excellent electrical and optical properties, which makes it a promising candidate for numerous electronic applications. Traditionally, ZnO thin films are deposited using a variety of processes, such as spin coating [29], spray analysis [30,31], pulsed laser deposition [32], molecular-beam epitaxy [33,34], atomic layer deposition [35,36] and RF sputtering [37,38,39]. A highest mobility of 300 cm^2^/V-s has been achieved [34] in Mg-doped ZnO heterostructures grown using molecular-beam epitaxy. This requires an ultra-high vacuum, leading to high cost. Alternatively, RF sputtering provides versatility, scalability, uniformity and high-quality thin films in a controlled environment even at low temperatures. This has paved the way for large-area and low-cost device applications.

Even though the mobilities of RF-sputtered ZnO thin films have reached the range of 70 cm^2^/V-s, defects in the ZnO thin films play a crucial role in realizing high-quality TFTs [40,41]. Various intrinsic defects such as oxygen vacancies, Zn interstitials, grain boundaries and dislocations are reported to play a major role in forming n-type semiconductors [42,43]. Among these, oxygen vacancies and Zn interstitials can act as shallow donors and/or deep acceptors depending on the charge state that would affect the optical and electrical properties. Advanced characterization techniques like X-Ray photoelectron spectroscopy (XPS), X-Ray diffraction (XRD) and UV–visible spectroscopy were employed to understand and manipulate the defects in thin-film optimization.

Singh S et al. reported a field-effect mobility of 0.6134 cm^2^/V-s and threshold voltage of 3.1 V in their ZnO TFT using RF sputtering with SiO_2_ as a gate insulator [38]. Similarly, Jong Hoon Lee et al. fabricated ZnO TFT with MgO as a gate insulator and reported a lower field-effect mobility of 0.0235 cm^2^/V-s, I_ON_/I_OFF_ ratio of ∼10^5^, threshold voltage of 2.2 V and sub-threshold (SS) value of 1.18 V/decade [39]. Brandon Walker et al. compared the performance of a ZnO TFT with various gate dielectric materials, namely, Al_2_O_3_, HfO_2_ and ZrO_2_, and achieved the highest on/off ratio of >10^5^ [44]. B. -S. Wang et al. achieved a mobility of 84.22 cm^2^/V-s and an I_ON_/I_OFF_ ratio of 3 × 10^6^ on a MgZnO/ZnO heterostructure TFT. It may be noted that the presence of defects such as O_2_ vacancies and Zn interstitials in ZnO significantly affects various electrical parameters, which are crucial for the realization of a normally off device (enhancement-type transistor) [45].

In this work, RF sputtering has been preferred over other techniques because of its non-equilibrium, large-area growth capability, and its ease of processing at low temperatures. Various properties of ZnO thin films were characterized by X-Ray diffraction, XPS, Hall measurements, SEM imaging and a UV–visible spectrophotometer. Hence, in this paper, the optimization of ZnO thin films has been systematically carried out using an RF sputtering technique with different argon:oxygen flow rates and different substrate temperatures. Based on the thin-film optimization, bottom-gate TFTs were fabricated and various device parameters such as the threshold voltage, I_ON_/I_OFF_ ratio, field-effect mobility and sub-threshold swing were investigated.

## 2. Materials and Methods

### 2.1. Deposition of ZnO Thin Films

ZnO thin films were deposited using RF magnetron sputtering on p-Si substrate with a resistivity of 5–10 Ω-cm. Prior to ZnO deposition, substrates were cleaned using RCA1 and RCA2 methods to remove organic and metal contaminations, followed by HF dip to remove native oxide [46]. During ZnO deposition, the target to substrate distance was maintained at 7.5 cm and the chamber was evacuated to 5.5 × 10^−6^ mbar. In order to maintain a contamination-free source material, the target was pre-sputtered for 10 min prior to the deposition. Further, a series of films were deposited at room temperature to optimize the Ar:O_2_ gas flow rate. Samples A, B and C represent 180 nm thick ZnO films deposited at different Ar:O_2_ flow rates, at a constant annealing temperature of 300 °C for 30 min in N_2_ ambient, as described in Table 1.

The gas flow rates were measured using the Aalborg mass flow controllers. Upon completion of the flow rate optimization, temperature optimization was carried out at room temperature (RT), 250 °C and 300 °C, as detailed in Table 1. Deposition rates of Samples D, C and E were determined to be 3 nm/min, 6 nm/min and 7 nm/min, respectively, using a Bruker profilometer (Bruker Nano Inc., Tucson, AZ, USA) (Figure S1).

Grazing incidence (GI) θ/2θ X-Ray diffraction was carried out on Samples A, B, C, D and E using a Rigaku Smartlab X-Ray Diffractometer (Rigaku corporation, Tokyo, Japan). Based on the FWHM values of the (002) XRD peak, various parameters such as the crystallite size, strain and dislocation density values were determined. Hall measurements were also carried out on the above samples at a magnetic field of 0.51 T using an Ecopia Van der Pauw HMS 3000 system (Ecopia, Anyang, Republic of Korea). Surface morphology of ZnO thin films was studied using a Zeiss ULTRA 55 scanning electron microscopy (Zeiss, OberKochen, Germany) (SEM) system on all the above samples. X-Ray photoelectron spectroscopy (XPS) analysis of the ZnO thin films was performed using a Kratos Axis Ultra spectrometer (Kratos Analytical, Manchester, UK), employing a monochromatic Al-Kα source. Absorption edges of all the above samples were derived from the transmission data measured in a UV-1650PC Shimadzu spectrophotometer (Shimadzu Corporation, Kyoto, Japan).

### 2.2. Device Fabrication and Characterization

Thin-film transistor devices with an optimized flow rate of 16:4 (Ar:O_2_) and 60 W RF power were fabricated on a single-side polished p-SiO_2_/Si (525 μm) substrate with an oxide thickness of 100 nm, procured from Prolyx Microelectronics Pvt., Ltd., Bengaluru, India. TFTs thus fabricated are named C1, D1 and E1 based on the variations in the process parameters, as listed in Table 2.

**Figure 1:**
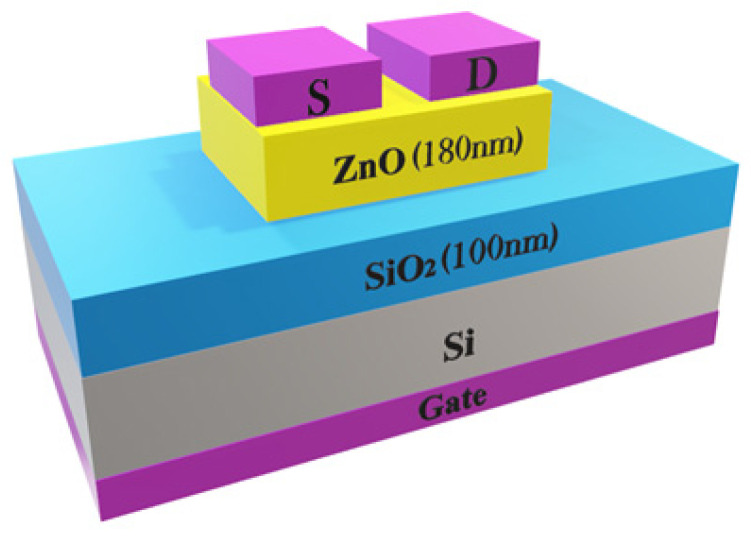
Schematic structure of ZnO TFT.

The mask for patterning the channel and electrode layer was printed on chrome glass using the mask writer (Figure S2). Exposure and patterning of the device were carried out on a Karl Suss MA6-BA6 mask aligner (SUSS Micro Tec., Garching, Germany). Figure 1 shows the schematic of the TFT device fabricated using a 2-step lithography process.

Firstly, 180 nm thick ZnO was deposited using RF sputtering, followed by the first lithography process wherein a 300 µm × 300 µm ZnO mesa structure was created by a wet etching process. Next, the second lithography was carried out to pattern the source and drain the electrodes. An amount of 100 nm Al metal was then deposited at room temperature using the thermal evaporation method, followed by a lift-off process, to obtain the desired source/drain (S/D) pattern. Finally, back-side SiO_2_ was selectively removed using HF dip, and blanket Al metal (100 nm) as the gate electrode was deposited using thermal evaporation. In order to improve the contact resistance, all the devices were annealed at 220 °C in N_2_ ambient for 10 min. Electrical properties of ZnO TFTs with a width to length (W/L) ratio of 50 µm/50 µm were examined using I–V transfer and output characteristics. I–V characteristics were carried out on a cascade Summit 11000B-M (Cascade Microtech, Beaverton, OR, USA) precision 4-axis semi-automated Probe station platform.

## 3. Results and Discussion

### 3.1. X-Ray Diffraction (XRD)

**Figure 2:**
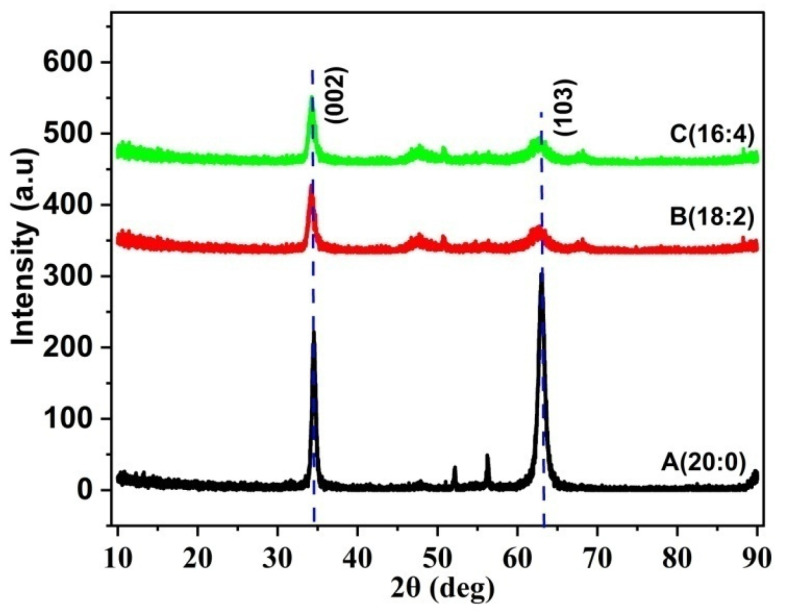
GIXRD spectra of ZnO thin-film Samples A, B and C with different Ar:O_2_ flow rates.

Figure 2 represents the grazing incident X-Ray diffraction of Samples A, B and C deposited at varying Ar:O_2_ flow rates. The XRD spectrum peaks match well with the data of JCPDS card no: 36-1451 [44]. Sample A showed a high-intensity (002) XRD peak at 34.53° and an even higher (103) peak at 62°. As the O_2_ ratio is increased in Samples B and C, the overall intensity of the ZnO (002) peak has decreased with the suppression of the (103) ZnO peak. The crystallite size, dislocation density and micro-strain were calculated using Equations (1)–(3) [47] and are listed in Table 3.

(1) $$D=\frac{0.9\lambda }{\beta Cos\theta }$$

(2) $$\delta =\frac{1}{D^{2}}$$

(3) $$\varepsilon =\frac{\beta }{4\tan\theta }$$

where λ is the wavelength of the X-Ray (1.54 Å), θ is the Bragg’s angle, β is the full width of half maximum (FWHM) of the XRD peak, δ is the dislocation density and D is the crystallite size.

**Figure 3:**
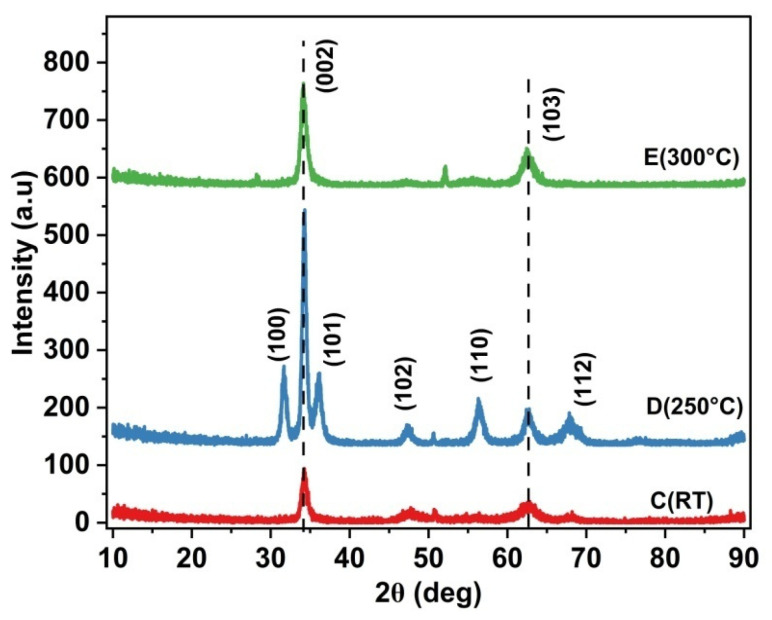
GIXRD spectra of samples with different substrate temperatures.

Figure 3 compares the GIXRD of Samples C, D and E deposited at RT, 250 °C and 300 °C, respectively. By maintaining the Ar:O_2_ flow rate at 16:4, Samples D and E exhibited notable improvement in the (002) XRD peak as compared to Sample C, indicative of improvement in the crystalline quality. All the samples except Sample A exhibited preferred orientation along the (002) plane. Hence, all our TFT devices were grown at the optimized gas flow ratio of 16:4 (Ar:O_2_) at room temperature, 250 °C and 300 °C. It may be noted that Sample D exhibits a polycrystalline property with distinct peaks at (100), (002), (101), (102), (110), (103) and (112).

Table 3 lists the crystalline size calculated from the FWHM values of the XRD spectrum. Sample A exhibited the lowest (002) XRD FWHM and highest crystalline size, whereas Sample C demonstrated the highest FWHM and lowest crystalline size. Even though Sample A exhibited the lowest strain in the microstructure, the presence of a dominant (103) XRD peak suggests that the surface is affected by the non-equilibrium growth condition of RF sputtering. At higher deposition temperatures of 250 °C and 300 °C in Samples D and E, the crystallite size increased to 13.85 nm and 11.38 nm, respectively. The reason for the increase in the crystallite size in Sample D could be explained by the fact that higher substrate temperatures assist in the surface migration of atoms/ions, leading to the appearance of an in-plane (100) XRD peak at 31° (Figure 3).

Figure S3 shows the conventional X-Ray diffraction spectra of Sample C, which exhibits a dominant (002) ZnO peak at 34°, and a relatively low-intensity (103) peak at 62°, indicative of preferential (002) crystal formation in the bulk layer. The intensity ratio of the (002)/(103) peak has been observed to increase from 3 in GIXRD to 9.52 in conventional XRD, indicative of c-axis orientation in the bulk layer. The sharp (002) peak in conventional XRD is evidence of c-axis orientation, and the relatively higher (103) peak in GIXRD is evidence of surface re-structuring during the final stages of RF sputtering. Yunlan Wang et al. [48] reported in their RF-sputtered ZnO thin films that the appearance of (103) GIXRD orientation is due to the native property of ZnO. With RF sputtering being a non-equilibrium growth technique, the re-orientation of atoms by diffusion may happen on the surface during the final stages of deposition, which could be the reason for the presence of the dominant (103) peak. Additionally, (103) XRD peaks are reported in ZnO thin films prepared by electro-deposition [49] and sol–gel [50] processes. It is observed that Sample A has the highest (103) peak intensity compared to any other sample. This could be attributed to the relatively low oxygen reactive species, enabling the Zn atoms to move freely along the surface. As the O_2_ flow rates are increased in Samples B, C, D and E, the influx of O_2_ species suppressed the free movement or diffusion of Zn atoms, leading to a low-intensity (103) peak. Hence, O_2_ over-pressure during the sputtering process becomes vital in depositing a smooth surface.

### 3.2. SEM

**Figure 4:**
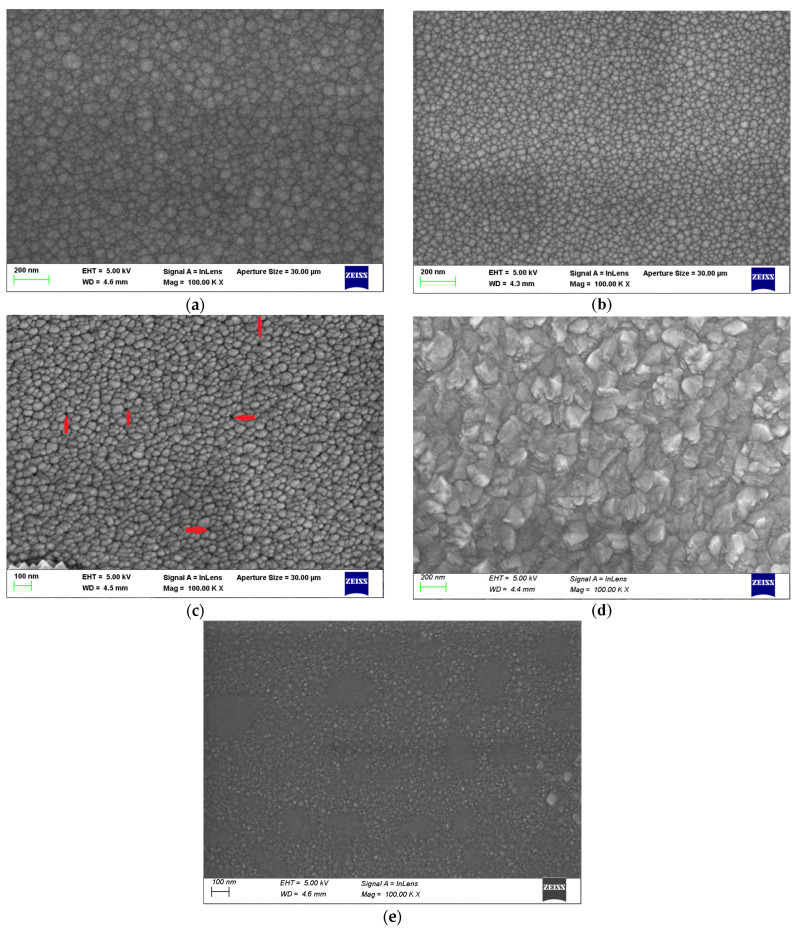
SEM image of ZnO thin-film Samples (**a**) A, (**b**) B, (**c**) C (Red arrows represents small voids between the crystallites), (**d**) D and (**e**) E.

Figure 4a–e show the SEM images of ZnO films A, B, C, D and E, respectively. Sample A, deposited at an Ar:O_2_ flow rate ratio of 20:0, shows a larger crystallite size with distinct contours. The presence of bigger grain crystalloids in the SEM image for Sample A corroborates the GIXRD data, revealing a crystallite size of 14.596 nm (Table 3). The crystallite size started to decrease with the highly dense surface morphology in Sample B. Both Samples A and B exhibit a granular and void-free surface with a high packing density. With a further increase in the O_2_ flow rate to 4 sccm in Sample C, structured morphology and isolated crystallites with the same size as Sample B have been observed. However, small voids between the crystallites started to appear in Sample C, as pointed out in Figure 4c. The voids disappear when the films are deposited at 250 °C (Sample D) and 300 °C (Sample E). In particular, the SEM image of Sample D (deposited at 250 °C) reveals a distinctly different surface morphology, possibly due to the migration of surface atoms forming larger crystallites. The larger contours corroborate with the multiple XRD peaks on the corresponding GIXRD spectrum (Figure 3), exhibiting both in-plane (100) and out-of-plane (002) peaks on the surface. Hence, the SEM image physically confirms that the sample deposited at 250 °C leads to the larger crystallite size of 13.858 nm, as extracted from the XRD data (Table 3).

The elemental mapping of the ZnO thin films deposited on Si was performed using the EDX. Figure S4 shows the presence of Zn and O_2_ species in all the deposited films. Table S1 lists the respective elemental compositions. It is well known that EDX scans deep into the silicon substrate; hence, XPS analyses were carried out to study the surface effects of ZnO and their related defects.

### 3.3. Optical Characterization

**Figure 5:**
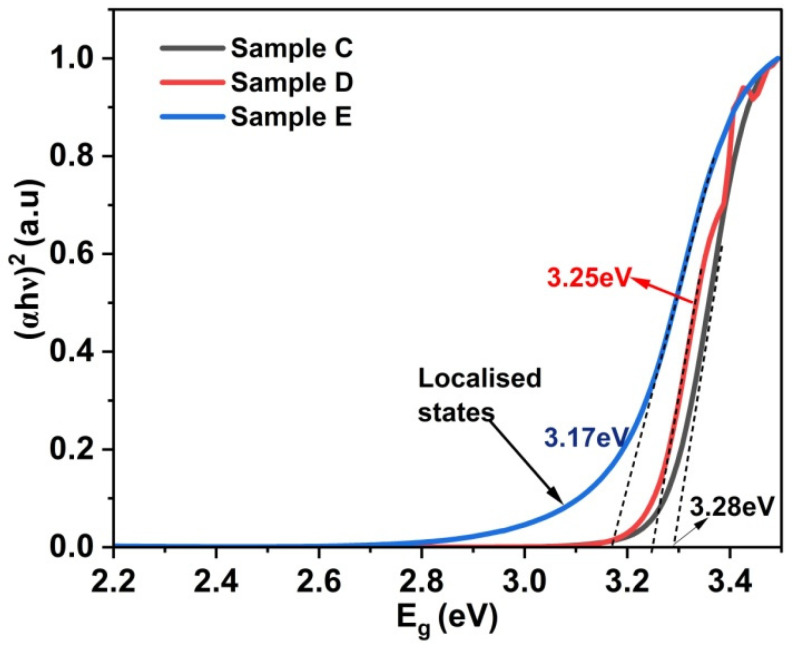
Tauc plot of Samples C, D and E.

The Tauc plot between (αhν)^2^ and the bandgap (E_g_) has been used to determine the absorption edge of the ZnO thin-film Samples C, D and E (Figure 5). All the Samples C, D and E show a bulk-like absorption edge, attesting to good-quality ZnO thin films. Sample C, deposited at RT, exhibits an absorption edge at 3.28 eV, whereas Sample D, deposited at 250 °C, shows a redshift in the bandgap of 3.25 eV. Sample E, deposited at 300 °C, shows a further redshift to 3.15 eV, which may be attributed to the increase in the crystallite size observed in the SEM image and XRD calculation [51].

Intrinsically, ZnO thin films have both oxygen vacancies and Zn interstitials exhibiting n-type conductivity [52]. The observed absorption spectra of our ZnO thin films can be separated into the following transitions: (i) ZnO band-to-band transition, (ii) transitions to localized band states (widely known as Urbach tails) and (iii) free carrier absorption [53]. There are several reports on Urbach tail states in ZnO thin films, due to structural disorders [54,55].

**Figure 6:**
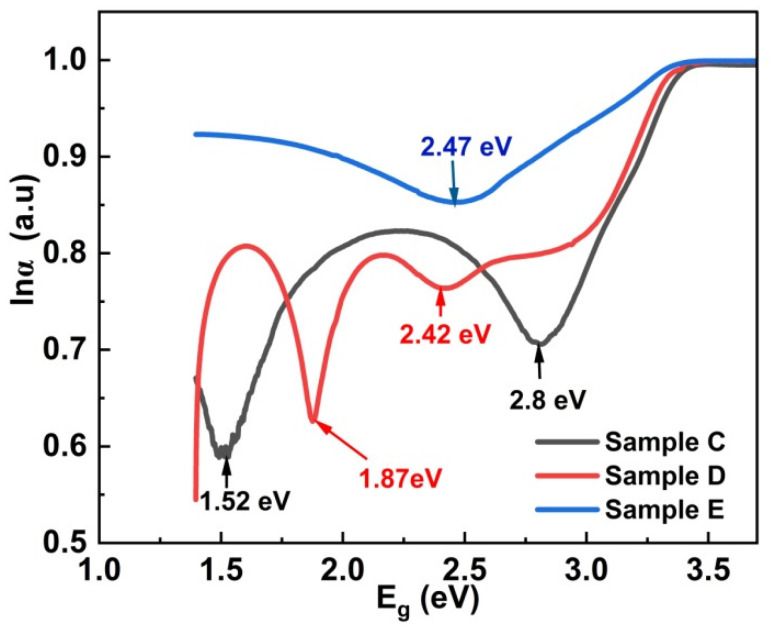
ln α vs. energy bandgap (E_g_).

In order to delineate various sub-bands of our samples, a natural log of the absorption edge and the bandgap was plotted [56]. Figure 6 shows the plot between ln (α) and the bandgap for Samples C, D and E. Sample C exhibits two Urbach tails at 2.8 eV and 1.52 eV, whereas Sample D exhibits a weak band-tail at 2.42 eV and a strong band-tail state at 1.87 eV. All the above Urbach tails observed in Samples C and D are attributed to the presence of both oxygen vacancies and Zn interstitials creating deep donor and acceptor levels within the bandgap, leading to strong sub-band absorption. There have been previous reports on ZnO nanoparticles exhibiting photoluminescence (PL) emission at around 2.8 eV, and this was attributed to the transition between Zn interstitials (I_Zn_) and the valance band. Our Sample C shows a similar Urbach tail at 2.8 eV; we attribute that to the presence of possible excess I_Zn_. The fast roll-off in Sample C becomes a slow roll-off in Sample D, with a reduced absorption coefficient, which could be due to the possible variation in localized I_Zn_. Additionally, Sample D exhibits a fast roll-off at 1.87 eV, which could be due to the possible transition between various localized defect states. Bandopadhyay et al. reported similar complexes of I_Zn_ below the conduction band (CB) and oxygen vacancies (V_O_) above the valance band (VB), with emissions at 1.7eV and 2 eV, respectively [57]. Hence, we speculate the presence of a sharp band-tail at 1.87 eV in Sample D is due to the transition between the I_Zn_ below the CB and V_O_ above the VB. These are highly localized band-tail states because of the sharp roll-off observed. Lastly, Sample E shows band-to-band absorption at 3.14 eV and a weak Urbach tail at 2.47 eV representative of possible (i) direct band transition and (ii) band-to-tail transition, respectively. It is worth noting that there is an overall reduction in the absorption coefficient in Sample E coupled with a slow roll-off at 2.47 eV. The absence of a sharp roll-off in Sample E may attest to the annihilation of localized defects; however, shallow defect states at 2.47 eV continue to exist.

### 3.4. XPS

**Figure 7:**
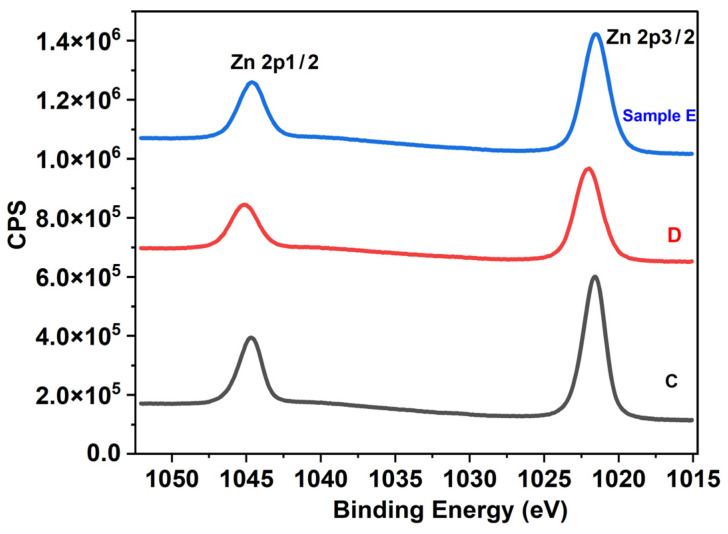
Core-level spectrum of Zn 2P peak.

Atomic % concentrations of the Zn and O_2_ elements were determined from the XPS survey spectrum for Samples C, D and E and are listed in Table 4. Sample C exhibits the lowest O_2_% (19.07%), which increases to 32.53% in Sample D and 44.83% in Sample E. Alternatively, the atomic Zn % starts to decrease as the samples are deposited at higher temperatures (Samples D and E). Sample E, deposited at 300 °C, is observed to reach the ideal ~50:50 stoichiometric ratio of ZnO. The presence of Zn^2+^ states bounded by O^2−^ states in all the samples is confirmed by the peak separation of 23.1 eV between the core levels, Zn 2p_1/2_ and Zn 2p_3/2_, as shown in Figure 7 [58].

**Figure 8:**
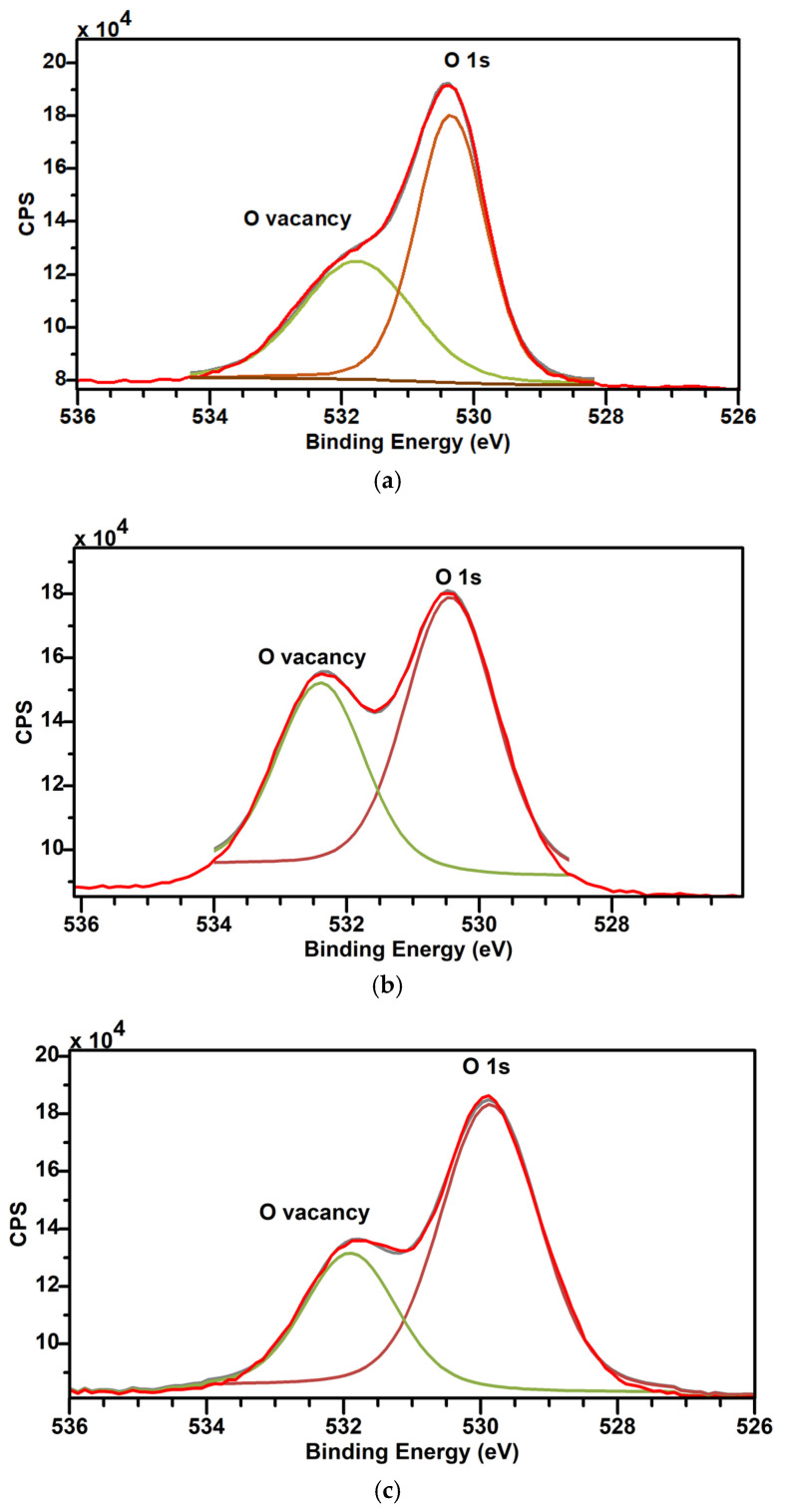
(**a**) XPS spectrum of O 1s peak—Sample C. (**b**) XPS spectrum of O 1s peak—Sample D. (**c**) XPS spectrum of O 1s peak—Sample E, with the following color representations: Red—Original XPS data, Green—O Vacancies component and Burgundy—O 1s peak component.

The O 1s XPS peaks of various films are shown in Figure 8a–c and their binding energies are listed in Table 5. The O 1s peak has been resolved into two peaks at ~530 eV and ~532 eV, representative of the O 1s peak and oxygen vacancies, respectively. Sample C exhibits a narrower O 1s peak at 530.3 eV, and a broader O_2_ vacancy peak at 531.7 eV. Sample C shows a lower area (58.62%) under the O 1s curve, indicative of a lower O_2_ concentration, as compared to Samples D (60.9%) and E (69.74%), which were deposited at higher temperatures (Table 1). It may be noted that Sample C underwent post-deposition annealing at 300 °C in N_2_ ambient that would have led to the desorption of O from the surface with a relatively low O_2_ atomic % of 19.07% (Table 4), thereby creating a spike in O2 vacancies (41.38%), as listed in Table 5. The relatively higher strain of 0.001% (Table 3) in Sample C also attests to the broader O_2_ vacancy peak (FWHM of 1.99 eV in Table 5). Alternatively, Samples D and E exhibit a relatively broader O 1s peak, and narrower O_2_ vacancy peaks. In particular, Sample D shows a broad O 1s peak with an FHWM of 1.6 eV, suggesting the presence of O_2_ complexes in addition to the O_2_ vacancies. Based on the XPS O 1s data, it is clear that Sample E exhibits better O_2_ incorporation, with the area under the O 1s curve being the highest at 69.74%.

**Figure 9:**
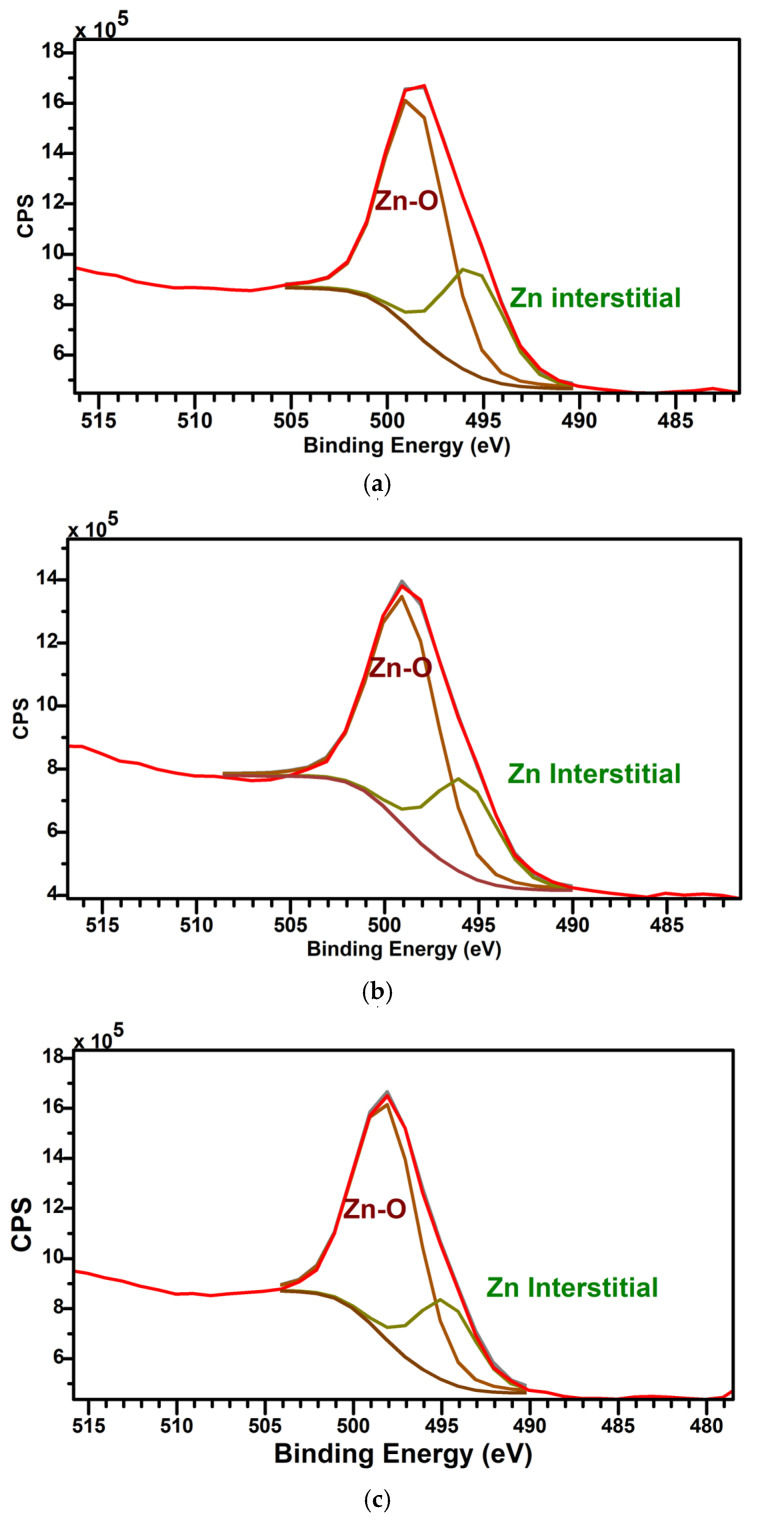
(**a**) Zn LMM Auger peak—Sample C. (**b**) Zn LMM Auger peak—Sample D. (**c**) Zn LMM Auger peak—Sample E, with the following color representations: Red—Original XPS data, Green—Zn interstitials component and Burgundy—Zn-O component.

Figure 9a–c illustrate the deconvoluted Zn LMM Auger peaks of Samples C, D and E. The concentrations of Zn interstitials (I_Zn_) are thus determined and listed in Table 6. The Auger peak at ~495 eV confirms the presence of I_Zn_ in the layers. Sample C, deposited at RT, exhibits a Zn-rich bulk with a high Zn atomic % of 73.56% (as illustrated in Table 4) and highest percentage of I_Zn_ (as listed in Table 6). A steady decrease in I_Zn_ has been observed as the deposition temperatures are increased to 250 °C and 300 °C (Samples D and E). We speculate that these I_Zn_ act as shallow donors, which become annihilated when the substrate temperatures are increased.

The voids in the SEM image (Figure 4c) and the presence of Urbach tails at 2.8 eV and 1.53 eV in the Tauc plot are clear evidence of high % I_Zn-_ and V_O_-related defects in Sample C due to room temperature deposition. Further annealing of Sample C at 300 °C also did not help in the annihilation of defects. Based on the XPS analysis, it is clear that higher deposition temperatures not only enhance the O_2_ incorporation, but also reduce I_Zn_ and V_O_ (oxygen-related defects). Auger analyses of the Zn LMM peak confirm the lowest percentage of I_Zn_ (24.23%) in Sample E.

**Figure 10:**
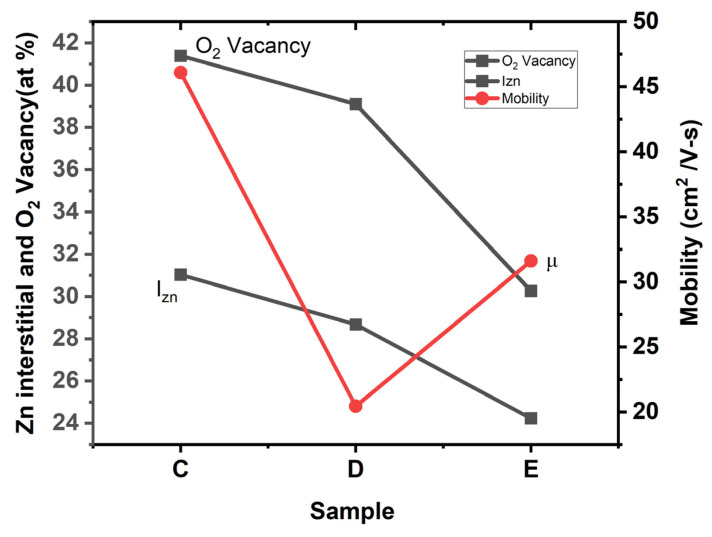
Plot of Zn interstitials, O_2_ vacancy and mobility of ZnO thin films.

Figure 10 shows the changes in the I_Zn_, V_O_ and mobilities with respect to the deposition temperatures (Samples C, D and E). It is evident that both the atomic % of I_Zn_ and V_O_ decreases as the deposition temperature increases. The changes in mobility can be explained in terms of variations in I_Zn_ and V_O_ in the lattice. All our Hall measurements showed that the films are n-type, attested to by the presence of V_O_ and I_Zn_, or both. Sample C exhibits the highest mobility, which is one of the highest reported in the literature (Table 7). The observed drop in the mobility from Sample C to D shall be explained by the relatively high % of I_Zn_ (28.67% in Sample D), which may act as scattering sites. The SEM images and XRD results also attest to the degradation layer quality in Sample D when deposited at 250 °C. The SEM image shows the coalescing nature of ZnO, denoting the wrinkled nature of the sample (Figure 4d), and the GIXRD spectrum shows the polycrystalline property on the surface (Figure 3).

Finally, Sample E, deposited at 300 °C, shows an increase in the Hall mobility to 31.6 cm^2^/V-s. This shall be explained by the reduction in I_Zn_ (24.23%) and improvement in Zn-O bonding (75.77%) (Table 6). The results of the UV–Vis spectrum also show a slow roll-off in the Urbach tail at 2.47 eV, confirming the annihilation of localized defect states, which further explains the improvement in mobility for Sample E. We speculate the absence of localized defect states assists the increase in the mobility of Sample E, whereas the presence of localized defects at 1.87 eV in Sample D acts as traps, resulting in a reduction in mobility.

### 3.5. Device Characterization

Thin-film transistors were fabricated on 100 nm SiO_2_/Si substrates with a ZnO channel layer. TFT Device C1 was fabricated at room temperature followed by 300 °C post-deposition annealing (similar to Sample C thin film), whereas Devices D1 and E1 represent TFTs deposited at 250 °C and 300 °C, respectively. All the TFTs exhibit n-type behavior, as represented by the I_D_–V_D_ curves. The drain current expressions for the enhancement-mode TFT are as follows:

Linear region

(4) $$I_{D}=\frac{W}{L}C_{ox}\mu_{FE}\left[\left(V_{GS}-V_{th}\right)V_{DS}-\frac{1}{2}V_{DS}^{2}\right]$$

Saturation region

(5) $$I_{D}=\frac{W}{2L}C_{ox}\mu_{FE}{\left(V_{GS}-V_{th}\right)}^{2}$$

Expression for field-effect mobility (μ_FE_):

(6) $$\mu_{FE}=\frac{g_{m}}{C_{ox}\frac{W}{L}V_{DS}}$$

(7) $$SS=\frac{{dV}_{GS}}{d\left(\log I_{D}\right)}$$

where W—width of the channel, L—length of the channel, C_ox_—capacitance per unit area and g_m_—transconductance.

#### 3.5.1. Drain Characteristics

**Figure 11:**
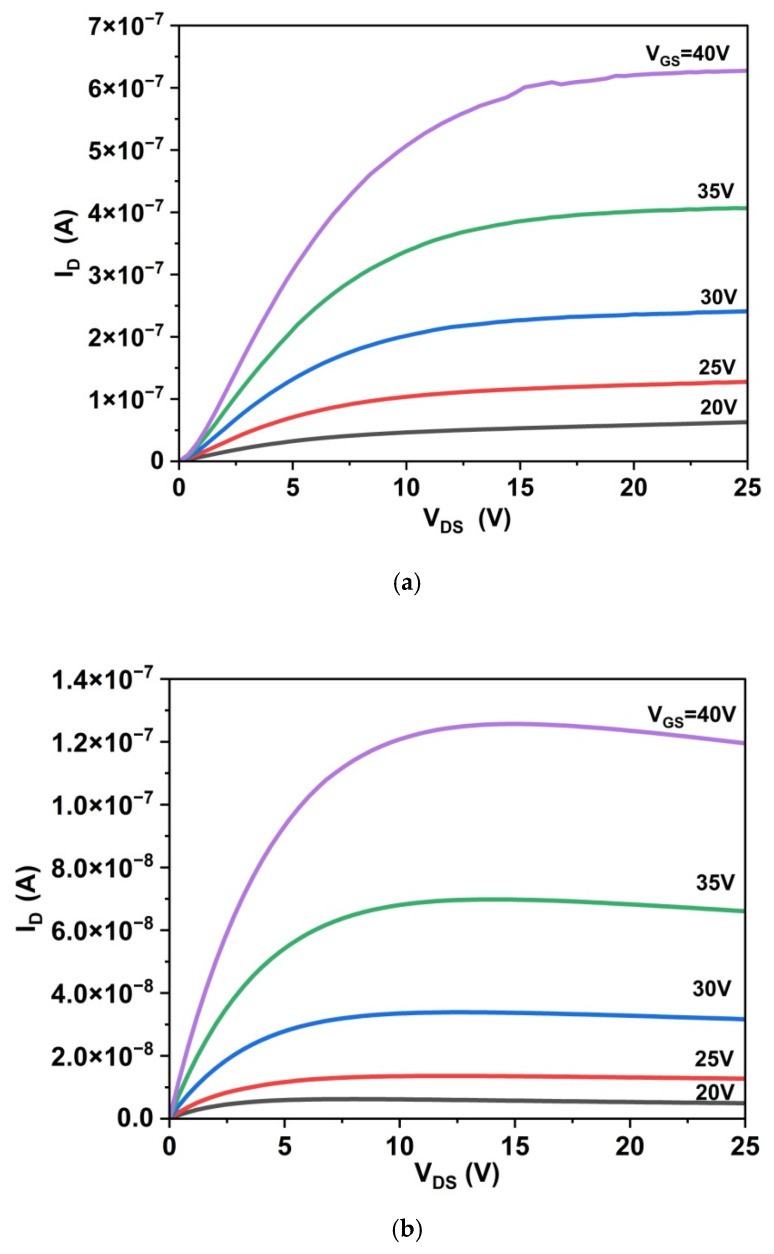

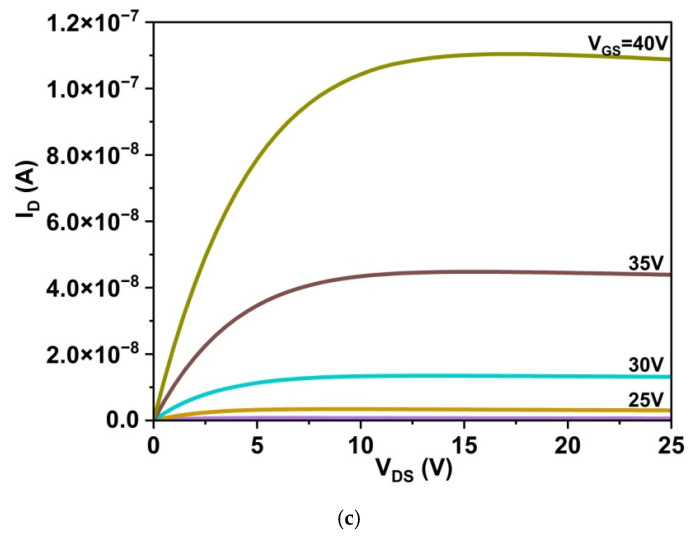
(**a**) Drain characteristics of TFT Device C1. (**b**) Drain characteristics of TFT Device D1. (**c**) Drain characteristics of TFT Device E1.

The drain characteristics were measured for TFT devices with the same channel length and width of 50 μm. The gate–source voltage (V_GS_) is varied from 0 to 40 V in steps of 5 V. At V_GS_ of 40 V, Device C1 reaches the highest drain current of 0.6 µA, whereas Devices D1 and E1 reach a maximum drain current of 0.11 µA. Variations in the drain currents match well with the variations in the bulk Hall mobility on Samples C, D and E, as represented in Table 8. Since the thickness of these devices is 180 nm, the bulk mobility dominates I_D_, where Sample C exhibits a highest Hall mobility of 46.09 cm^2^/V-s, corroborating with the high drain current of 0.6 µA in Device C1 (Figure 11a).

#### 3.5.2. Transfer Characteristics

**Figure 12:**
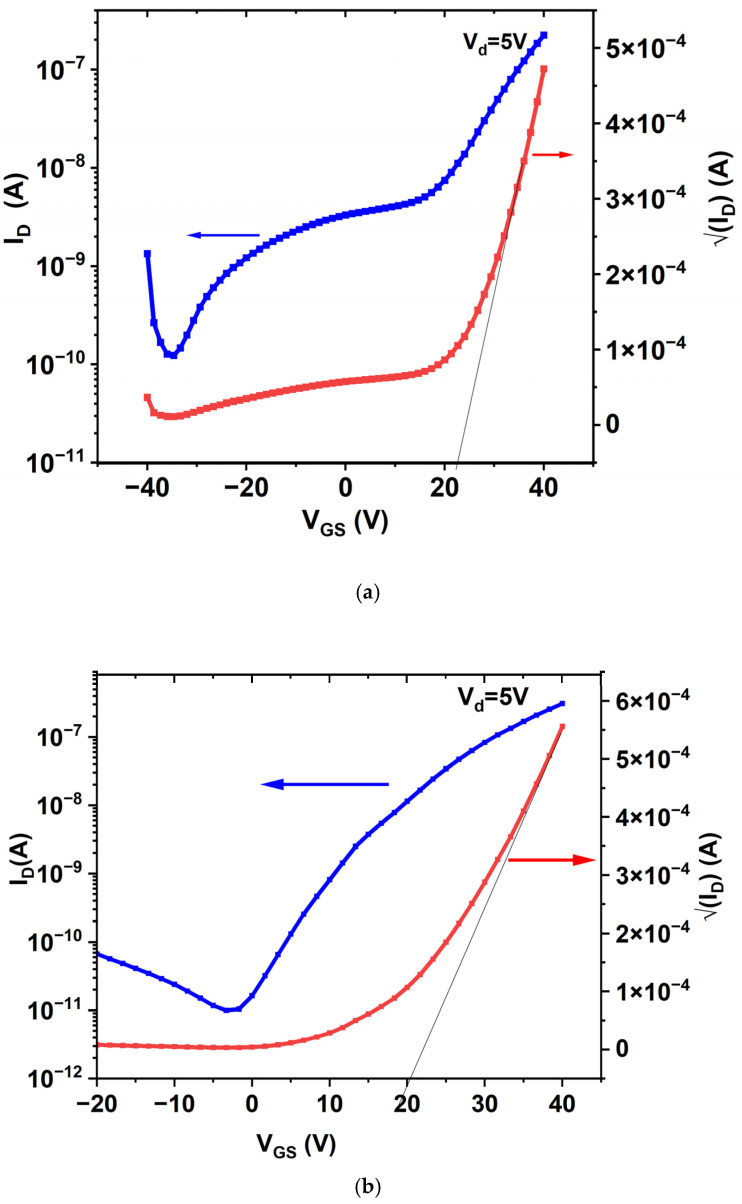

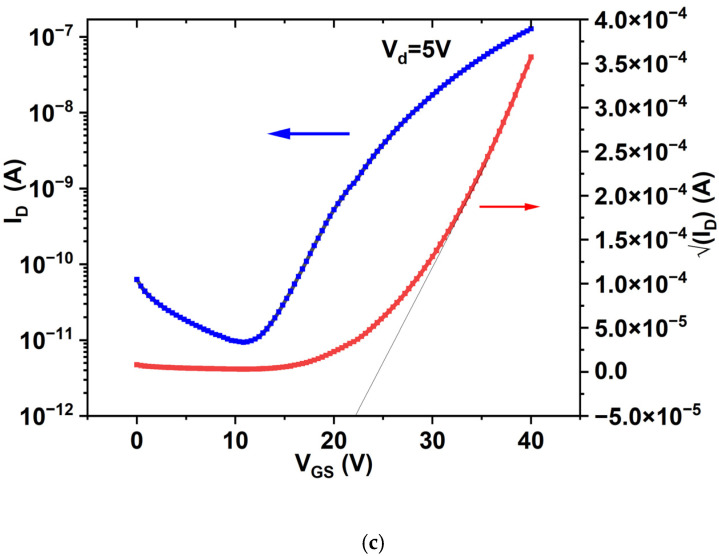
(**a**) Transfer characteristics of TFT Device C1. (**b**) Transfer characteristics of TFT Device D1. (**c**) Transfer characteristics of TFT Device E1.

Figure 12a–c represent the transfer characteristics of the above TFT Devices C1, D1 and E1 at V_DS_ = 5V. The gate voltage (V_GS_) is swept from −40 V to +40 V. The effect of the deposition temperature on the transfer curves shows that all the TFTs operate in the enhancement mode. Table 8 displays the electrical parameters of TFTs deposited at different temperatures. The I_OFF_ current for Device C1 is of the order of nanoamperes (~1.2 × 10^−10^ A), indicative of a leaky device. The presence of voids between the crystallites observed in the SEM image, a smaller crystallite size (10.94 nm) and grain boundaries could be the reason for the nanoampere range leakage current in Sample C. Even though the bulk Hall mobility is determined to be the highest in Sample C, we speculate the high carrier concentration of 2.6 × 10^17^ cm^−3^ is predominantly defect-induced transport. The defect is confirmed by the presence of two localized band-tail states in the absorption data (Figure 6) at 1.53 eV and 2.8 eV. As the deposition temperature is increased to 250 °C and 300 °C in Devices D1 and E1, respectively, the leakage current substantially reduces to picoamperes of 9.96 × 10^−12^ A and 9.35 × 10^−12^ A, respectively.

Our thin-film investigation based on GIXRD and SEM analyses suggests that surface modifications happen when samples are deposited at higher temperatures of 250 °C and 300 °C. It may be noted that Devices D1 and E1 were subjected to oxygen over-pressure for ~2 h prior to breaking the vacuum. During the ramping down of the deposition temperature to RT, we speculate the surface layer is modified due to chemisorption of O_2_ atoms. The effect of chemisorption is also confirmed in our GIXRD data, where the intensity of the ZnO (103) peak is increased in Samples D and E. Our XPS analyses also support the theory of O_2_-rich layers in Samples D and E, where relatively high O 1s peaks are observed. Hence, the surface modification due to chemisorption is strongly believed to increase the crystallite size (13.83 nm and 11.38 nm) and reduce the grain boundaries and oxygen vacancies, which are combined indications of the reduced defects in high-temperature-deposited TFTs (D1 and E1).

The turn-ON voltage (V_ON_) for Device C1 is measured at −34.6 V, and positively shifts to −3.33 V and 10.8 V in Devices D1 and E1, respectively. The decrease in I_OFF_, increase in V_ON_ voltage and high I_ON_/I_OFF_ ratio are clear evidence of the improved switching property for Devices D1 and E1 (Table 8). The shift in the V_ON_ is attributed to O_2_-rich thin films deposited at 300 °C, as also confirmed by the high O1s peak in the corresponding XPS spectrum (Figure 8a–c). Sangwon Lee et al. [72] similarly claimed in their InGaZnO TFTs that a shift in the turn-ON voltage is due to O_2_-rich layers.

Table 8 lists the field-effect mobility (µ_FE_) and threshold voltage (V_Th_) values of Devices C1, D1 and E1. In general, the field-effect mobility and threshold voltage are inversely related in a ZnO deposition [73]. Device C1 exhibits the highest V_Th_ of 23.1 V and lowest μ_FE_, whereas Devices D1 and E1 exhibit a lower threshold voltage and higher field-effect mobility as compared to Device C1. Our XPS data confirm a higher incorporation of O_2_ at 32.53% and 44.84% in Devices D1 and E1, respectively, which explains our claim on V_Th_ and μ_FE_. In addition, the microstructure analyses of the SEM images show the presence of voids, which may act as charge trapping regions affecting the threshold voltage of Device C1. Further, a larger crystalline size of 13.858 nm and 11.385 nm in Devices D1 and E1, respectively, (Table 3) corroborating the larger crystallite size in the SEM images (Figure 4), explains the drop in the threshold voltage (V_Th_) and increase in the field-effect mobility (μ_FE_).

Another parameter that defines the TFT performance is a sub-threshold swing (SS), which shows a decreasing trend from RT to high-temperature deposition. We propose the reason for the high SS in Sample C1 is due to O_2_ deficiency, leading to a nanoampere range of the leakage current. As the deposition temperature is further increased, O_2_-rich films in Devices D1 and E1 are the plausible reason for the lower leakage current, leading to lower values of SS.

#### 3.5.3. Variation in ZnO Layer Thickness

**Figure 13:**
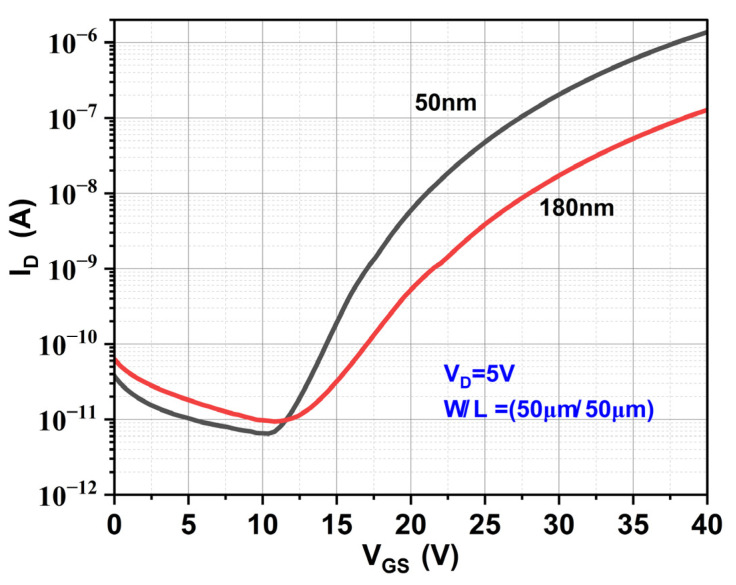
Transfer characteristics of ZnO TFT with variation in channel layer thickness.

Figure 13 describes the transfer characteristics of ZnO TFTs with varying channel thicknesses of 180 nm and 50 nm, which represent Devices E1 and E2, respectively. Table 9 lists the variation in the TFT parameters for the 180 nm and 50 nm thick ZnO active layers. All other process parameters are unchanged except the ZnO thickness. Device E2 exhibits a lower leakage current compared to Device E1, which could be explained by the channel thickness being less than the width of the depletion layer, which was calculated using the following relation.

(8) $$d_{ch}<W_{dep}=\sqrt{\frac{{4\varepsilon }_{0}\varepsilon_{r}\varphi_{b}}{qN_{e}}}$$

where d_ch_—channel thickness, W_dep_—depletion width, ε_r_—relative permittivity of ZnO, φ_b_—for the potential gap between the Fermi level and intrinsic level, q—electron charge and N_e_—carrier concentration.

A reduction in the channel thickness has increased the I_ON_ by 10-fold, leading to the improvement in the I_ON_/I_OFF_ ratio from 10^4^ to 10^5^. Consequently, the reduction in the sub-threshold slope is also observed in the thinner Device E2.

## 4. Conclusions

In conclusion, ZnO thin films deposited using RF sputtering were systematically optimized by varying the Ar:O_2_ gas flow rates and deposition temperatures. Structural, optical, microstructural and electrical properties were studied using XRD, a UV–visible spectrophotometer, XPS, Hall measurements and SEM imaging. The XRD results revealed polycrystalline properties on all the thin films. The UV–visible spectroscopy data showed a bulk-like absorption edge ranging from 3.28 eV to 3.17 eV, in addition to Urbach tail states within the bandgap. Thin films deposited at room temperature and annealed in N ambient resulted in low O_2_ incorporation, high oxygen vacancies and high Zn interstitials. From the absorption spectrum, we conclude that defects are highly localized in Sample C. Even though the films are deposited in an O_2_-rich environment, annealing in N ambient resulted in the desorption of O_2_ atoms from the surface, evident by the presence of voids in the corresponding SEM image. The Hall measurement derives a highest carrier mobility of 46.09 cm^2^/V-s in Sample C deposited at RT, possibly due to the high % of Zn interstitials. Deposition at 250 °C resulted in a polycrystalline top layer from the GIXRD, reduced % of oxygen vacancies and Zn interstitials confirmed by the XPS data and reduced band-tail states in the corresponding absorption spectrum, which explains the reduction in Hall mobility to 20.43 cm^2^/V-s. A further increase in the deposition temperature to 300 °C resulted in the overall improvement in the layer quality, attested to by the increase in the crystallite size from the XRD and SEM analyses, improvement in O_2_ incorporation and reduction in O_2_ vacancies and Zn interstitials from the XPS results, and substantial reduction in the localized Urbach tail from the absorption spectrum. Hence, based on the above analyses, ZnO thin film deposited at 300 °C seems to exhibit the best results even though a bulk mobility of 31.6 cm^2^/V-s has been obtained on Sample E.

Thin-film transistors with a W/L dimension of 50/50 µm were subsequently fabricated at RT, 250 °C and 300 °C. The device fabricated with RT deposition and 300 °C annealing resulted in a high leakage current (1.2 × 10^−10^ A) and high carrier concentration of 2.6 × 10^17^ cm^−3^, which are predominantly due to defect-induced transport. However, TFTs deposited at higher substrate temperatures of 250 °C and 300 °C showed a reduction in the leakage current (9.96 × 10^−12^ A), threshold voltage (20.3 V), sub-threshold swing (4.43) and increase in the I_ON_/I_OFF_ ratio (10^4^). A reduction in the ZnO layer thickness in the TFT at 300 °C ensured an O_2_-rich surface through chemisorption, leading to a reduction in the leakage current to 6.87 × 10^−12^ A and sub-threshold swing to 2.8 V. Thus, these improvements in the TFT performance are governed by precise deposition process control such as the deposition temperature, ZnO layer thickness, towards the improvement in the crystalline quality, reduction in oxygen vacancies and Zn interstitial-related defects. From the above analyses, our optimal process parameter results have been obtained on 50 nm thick TFTs with a W/L dimension of 50/50 µm deposited at 300° C.

## Figures and Tables

**Table 1:** Deposition parameters of ZnO thin films.

Sample	Deposition Temperature	Ar:O_2_ Flow Rate	Annealing Temperature and Duration	RF Power
A	RT	20:0	N_2_ ambient 300 °C, 30 min	60 W
B	RT	18:2		
C	RT	16:4		
D	250 °C	16:4	-------	60 W
E	300 °C	16:4		

**Table 2:** Deposition parameters and Hall performance of ZnO TFT.

Sample	Deposition Temperature	Annealing Temperature and Ambient	Ar:O_2_ Flow Rate	Post-Contact Annealing	Carrier Concentration (cm^−3^)
C1	RT	300 °C in N_2_ ambient	16:4	220 °C	2.6 × 10^17^
D1	250 °C	-			1 × 10^16^
E1	300 °C	-			1.6 × 10^17^

**Table 3:** ZnO thin-film parameters extracted from XRD.

Sample	FWHM (β) (Radians)	2θ (Deg)	Crystallite Size D (nm)	Dislocation Density δ (Lines/m^2^)	Lattice Parameter Spacing (nm)	Micro-Strain ε
A	0.57	34.53	14.596	0.0047	0.260	0.0008
B	0.7	34.25	11.876	0.0071	0.262	0.0009
C	0.76	34.3	10.940	0.0084	0.261	0.0010
D	0.6	34.31	13.858	0.0052	0.261	0.0008
E	0.73	34.15	11.385	0.0077	0.262	0.0010

**Table 4:** Atomic % calculation from XPS survey spectrum.

Element	C	D	E
O 1s	19.07	32.53	44.84
Zn 2p	73.56	56.76	47.66
C	7.36	10.71	7.5

**Table 5:** Oxygen vacancies obtained from XPS.

Sample	O_2_ Vacancy			O 1s Peak		
	Binding Energy (eV)	% Conc	FWHM	Binding Energy (eV)	FWHM	% Conc
C	531.77	41.38	1.99	530.354	1.25	58.62
D	532.38	39.1	1.54	530.43	1.6	60.9
E	531.88	30.26	1.57	529.88	1.7	69.74

**Table 6:** Deconvolution and % concentration of Zn-O and Zn interstitials from Zn Auger LMM peak.

	C	D	E
Zn-O	68.97	71.33	75.77
Zn Interstitials	31.03	28.67	24.23

**Table 7:** Comparison of ZnO thin-film electrical properties with the literature.

Year	Deposition Method	Mobility (cm^2^/V-s)	Carrier Concentration (cm^−3^)	Resistivity (Ω-cm)
2017 [59]	Sputtering	17.3	2.3 × 10^18^	-
2015 [60]	Sputtering	24.17	6.21 × 10^15^	-
2020 [61]	Pulsed Laser Deposition	34.6	2.28 × 10^18^	0.0792
2020 [62]	Atomic Layer Deposition	17.36	4.32 × 10^20^	8.33 × 10^–4^
2020 [63]	Sol–gel	0.05	1.2 × 10^15^	-
2020 [64]	Sputtering	18	-	3 × 10^–4^
2014 [65]	Sputtering	0.064	1.4 × 10^19^	6.8
2011 [66]	Sputtering	34	9.29 × 10^11^	1.98 × 10^5^
2011 [67]	Spray pyrolysis	0.26	7.27 × 10^15^	3.20 × 10^3^
2011 [40]	Sputtering	8.9	4.2 × 10^16^	31.8
This work	Sputtering	46.09	2.6 × 10^17^	1.96
TFT				
2018 [46]	Sputtering	0.7 *	-	-
2018 [68]	Sputtering	0.62 *	-	-
2020 [69]	Plasma ALD	0.782 *	-	-
2023 [70]	ALD	3.1 *	-	-
2023 [71]	Sputtering	0.08 *	-	-
This work	Sputtering	1.1 *	-	-

* Field-effect mobility of TFT.

**Table 8:** Electrical parameters of TFTs deposited at different temperatures.

Sample	Bulk Mobility (cm^2^/V-s)	I_ON_ (μA)	V_ON_ (V)	V_Th_ (V)	SS (V/dec)	I_ON_/I_OFF_ Ratio	μ_FE_ (cm^2^/V-s)
C1	46.09	0.223	−34.6	23.1	30	10^3^	0.062
D1	20.43	1.58	−3.33	20.3	6.61	10^4^	0.64
E1	31.6	0.127	10.8	21.7	4.43	10^4^	0.10

**Table 9:** Electrical parameters of ZnO TFT with variation in active layer thickness.

Device	V_ON_ (V)	V_Th_ (V)	SS (V/dec)	I_ON_/I_OFF_ Ratio	μ_FE_ (cm^2^/V-s)
E1 (180 nm)	11.2	21.7	4.43	10^4^	0.10
E2 (50 nm)	10.4	23.29	2.8	10^5^	1.1

## Data Availability

The original contributions presented in the study are included in the article/Supplementary Materials, further inquiries can be directed to the corresponding author.

## Supplementary Materials

The following supporting information can be downloaded at: https://www.mdpi.com/article/10.3390/ma17215153/s1, Figure S1: thin-film thickness measurement using Profilometer; Figure S2: image of the dark-field photomask used for channel creation and light-field photomask used for contact metallization; Figure S3: conventional XRD of ZnO thin film deposited at RT with different Ar:O2 flow rate; Figure S4: EDX analysis of ZnO thin films of Samples A, B, C; Table S1: elemental composition analysis of ZnO thin films.
