# Supplementary material for: Effect of Deposition Temperature on Zn Interstitials and Oxygen Vacancies in RF-Sputtered ZnO Thin Films and Thin Film-Transistors
Source: Materials (Basel). 2024 Oct 23;17(21):5153. doi: 10.3390/ma17215153 (PMC11547206; doi:10.3390/ma17215153)
Supplement: Supplementary file 1 [file materials-17-05153-s001.zip › materials-3244279-supplementary.pdf]

# Supplementary Information

# Effect of Deposition Temperature on Zn Interstitials and O Vacancies in RF sputtered ZnO Thin films and TFTs

Sasikala Muthusamy[1], Sudhakar Bharatan[1]*, Sinthamani Sivaprakasam[1] and Ranjithkumar Mohanam[1]

[1] Department of Electrical and Electronics Engineering, Sri Venkateswara College of Engineering, Chennai, India.

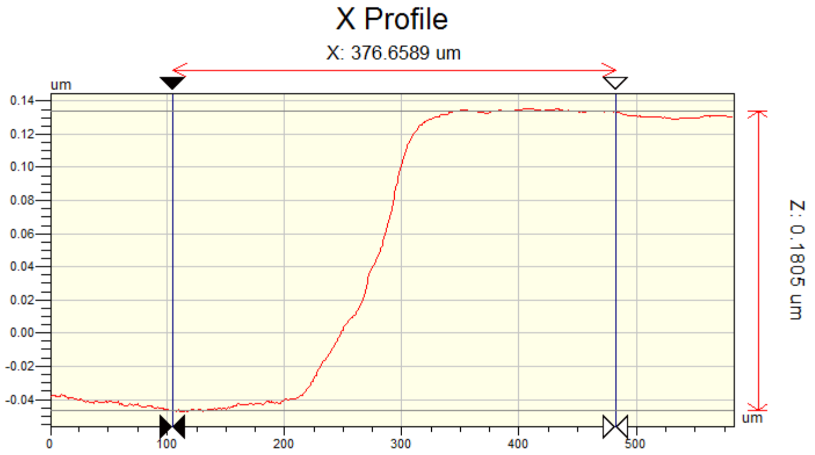


**Figure.S1:** Thin film thickness measurement using Profilometer

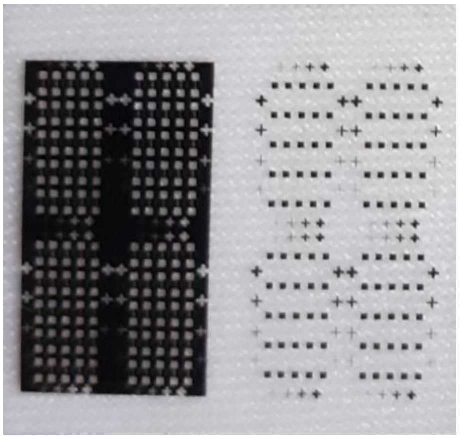

**Figure.S2**: Image of the dark field photomask used for channel creation and light field photomask used for contact metallization.

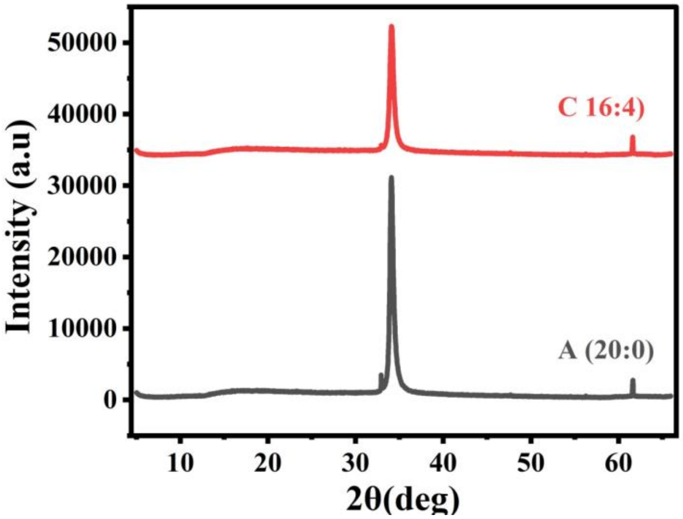


**Figure.S3:** Conventional XRD of ZnO thin film deposited at RT with different Ar:$O_2$ flow rate

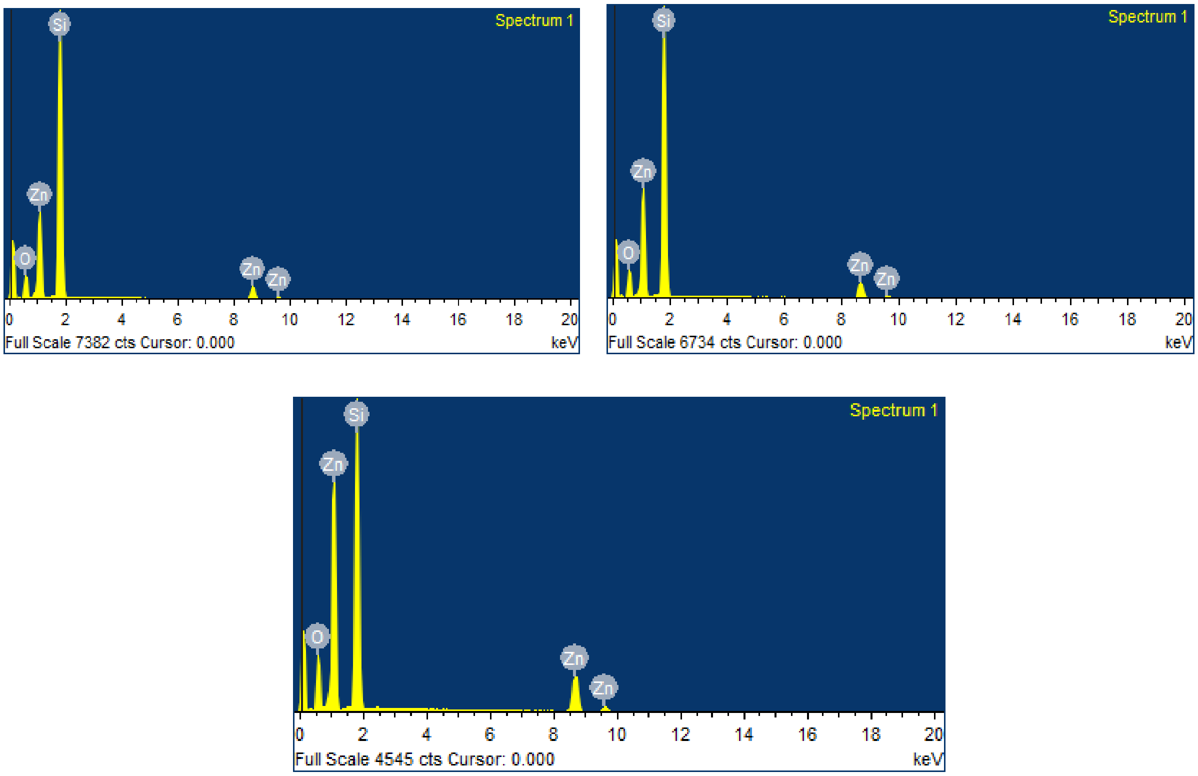


**Figure.S4**: EDX analysis of ZnO thin films of Samples A, B, C

**Table. S1:** Elemental composition analysis of ZnO thin films

| Element | Atomic percentage | | |
|---|---|---|---|
| | A | B | C |
| O | 42.56 | 37.51 | 35.95 |
| Zn | 16.37 | 10.69 | 9.02 |
